# Sebaceous carcinoma of trunk with bilateral axillary lymph node metastasis: a rare presentation of malignant adnexal tumor in young adult

**DOI:** 10.1093/jscr/rjac280

**Published:** 2022-06-14

**Authors:** Lokesh Shekher Jaiswal, Durga Neupane, Nimesh Lageju, Sarada Khadka, Bijay Sah, Anju Pradhan

**Affiliations:** Department of Surgery, B.P. Koirala Institute of Health Sciences, Dharan, Nepal; Department of Medicine, B.P. Koirala Institute of Health Sciences, Dharan, Nepal; Department of Medicine, B.P. Koirala Institute of Health Sciences, Dharan, Nepal; Department of Surgery, B.P. Koirala Institute of Health Sciences, Dharan, Nepal; Department of Surgery, B.P. Koirala Institute of Health Sciences, Dharan, Nepal; Department of Pathology, B.P. Koirala Institute of Health Sciences, Dharan, Nepal

## Abstract

Malignant adnexal tumors of skin are rare. Sebaceous carcinoma—a rare and aggressive cutaneous tumor—is frequently located in the periorbital region. Malignant adnexal tumors of sebaceous carcinoma type on trunk with axillary lymph node and distant metastasis in young adult is rare. The incidence of extraocular sebaceous carcinoma has been expected to be 0.06 per 100 000 person-years with an increased incidence in elderly patients and men. With a note of mimicking benign dermatologic conditions, definitive diagnosis of sebaceous carcinomas is often hindered, increasing morbidity and mortality for patients. Herein, we present a case of a 35-year-old man with a large ulcero-proliferating lesion of trunk region previously resected for a small swelling and eventually presented with the recurrent lesion and diagnosed as sebaceous carcinoma of trunk with bilateral axillary lymphnode and distant bone metastasis demonstrating several diagnostic and management challenges.

## INTRODUCTION

Malignant adnexal tumors of skin (MATS) are rare. A retrospective study of the patients registered in the surveillance, epidemiology and end results (SEER) database in the USA from 1988 to 2006, found 4032 MATS of which only 16.8% were present in trunk and only 7.4% demonstrated lymph node metastases [1]. The age-adjusted incidence rate for MATS is 5.1 per 1 million person-years. Sebaceous carcinoma—a rare and aggressive cutaneous tumor—is frequently located in the periorbital region [2, 3]. Roughly 25% of cases of sebaceous carcinoma are extraocular with a focus in the head and neck region [4]. The incidence of extraocular sebaceous carcinoma is 0.06 per 100 000 person-years with an increased incidence in elderly patients and men [2]. With a note of mimicking benign dermatologic conditions, definitive diagnosis of sebaceous carcinomas is often hindered, increasing morbidity and mortality for patients.

Herein, we present a case of a 35-year-old man with a large ulcero-proliferating lesion of trunk, medial to left scapula near spine previously resected for a small swelling over the same region and eventually diagnosed as sebaceous carcinoma of trunk with bilateral axillary lymph node and distant bone metastasis demonstrating several diagnostic and management challenges.

## CASE PRESENTATION

**Figure 1:**
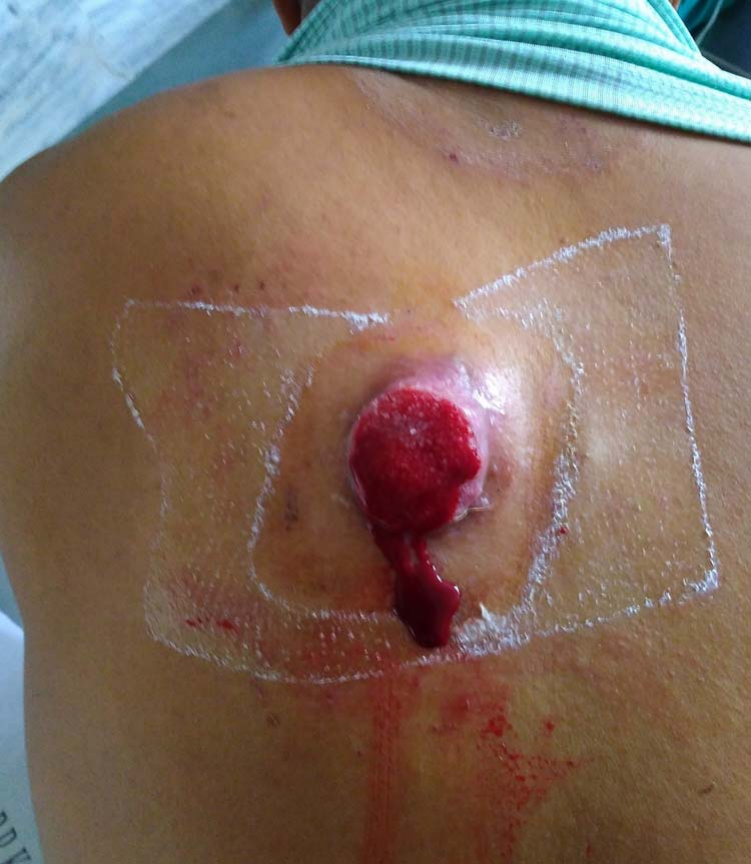
A large ulcero-proliferating lesion in back medial to left scapula near midline.

**Figure 2:**
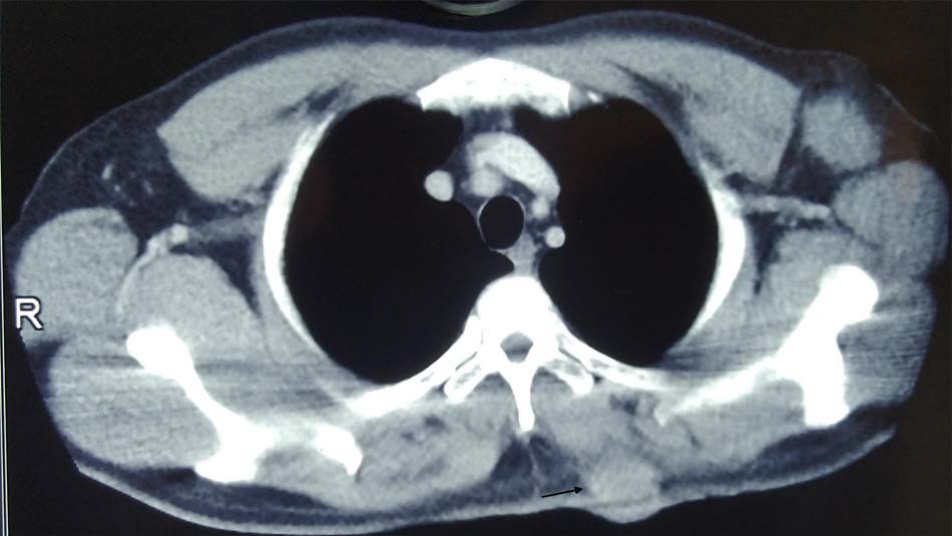
CT image showing well defined lesion in skin and subcutaneous tissue (arrow).

**Figure 3:**
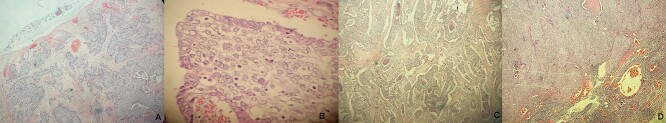
(**A**) Photomicrograph (H&E, ×10) showing proliferation of tumor cells in lobules, nest, trabeculae and tubules in the dermis. (**B**) Microscopic examination (H&E, ×20) showing tumor cells with high nucleo-cytoplasmic ratio, round to ovoid nuclei, irregular nuclear membrane and moderate amount of clear vacuolated cytoplasm. (**C**) Comedonecrosis pattern (H&E, ×10). (**D**) Microscopic examination showing lymphnode metastasis (H&E, ×10).

A 35-year-old male presented with large fungating lesion over his back. It was 8-cm round ulcero-proliferating lesion medial to left scapular region near spine (Fig. 1). On examination there were palpable left axillary lymph nodes. He had a history of small swelling over the same region, which was excised by local practitioner 2 months back. The details of the procedure and histopathology were not available. He is non-smoker, non-alcoholic with no history of malignancy in family. Tissue biopsy from lesion and fine needle aspiration cytology (FNAC) of left axillary lymph node side was positive for carcinoma. Contrast enhanced computed tomogram (CECT) of chest and abdomen was done. CECT showed well-defined lesion in skin and subcutaneous layer medial to left scapular region near spine (Fig. 2). There was no evidence of malignancy or metastasis to other organs in chest and abdomen. Colonoscopy, upper gastrointestinal endoscopy, CT head and ultrasound of bilateral breast was normal. FNAC of subcentric right axillary lymph node was also positive for metastasis. Therefore the diagnosis of malignant cutaneous adnexal tumor with bilateral axillary metastasis was made. He underwent wide local excision of the lesion with 2-cm margin and modified rhomboid flap coverage along with bilateral axillary dissection. Histopathology of the lesion confirmed the malignant adnexal tumor with closest resemblance to sebaceous carcinoma (Fig. 3). The margins were free of tumor cells. On immunohistochemistry examination, the tumor was positive for Her-2, GATA-3, CK-7 and EMA. It was negative for S-100, CEA, ER, PR, AR, TTF-1 PAX-8, CDX-2 and CK-20. He received radiotherapy for locoregional control. Six months later, he again presented with severe back ache. Magnetic resonance imaging was suggestive of bone metastasis. Medical oncological consultation was sought and received six cycles of Docetaxel and Cisplatin based chemotherapy. After 3 months of follow-up he is asymptomatic.

## DISCUSSION

Cutaneous adnexal tumors are a large group of benign and malignant lesion that exhibit morphologic differentiation towards one of the four primary adnexal structures present in the normal skin: hair follicles, apocrine gland, eccrine gland and sebaceous glands.

Sebaceous carcinomas frequently look like molluscum contagiosum, pyogenic granuloma, keratoacanthoma and squamous cell carcinoma [5]. Given the diverse clinical presentation and asymptomatic growth period, diagnosis and treatment of extraocular sebaceous carcinoma is often delayed [6]. Diagnosis necessitates pathologic confirmation of neoplastic cells with sebaceous differentiation, which can frequently be done by conventional microscopic methods. These common features include vacuolated cytoplasm, high mitotic activity and nuclear pleomorphism [7]. However, immunohistochemistry often helps in cases with poor differentiation or less obvious findings to avoid confusion with other dermatologic malignancies such as basal cell and squamous cell carcinoma [7, 8]. In our case, the microscopic histopathological and immunohistochemistry examination was suggestive of sebaceous carcinoma.

Despite the fact that extraocular sebaceous carcinoma is typically regarded as less aggressive than its ocular counterpart, extraocular tumors have also been reported in the literature to metastasize regionally and rarely to distant sites [9]. Older case series have conveyed rates of nodal disease as high as 21% [10]. However, in a retrospective review from 2009 of 1349 cases of sebaceous carcinoma, both ocular and extraocular, 1.7% of patients had clinical or pathologic evidence of lymph node involvement and 5.3% of patients received radiation therapy [2]. In our case, there was bilateral axillary lymph nodes metastasis and distant metastasis to bone which is extremely rare in young adult in thirties.

Wide local excision with minimum 1-cm peripheral margin down to the deep fascial plane is recommended for extraoccular sebaceous carcinoma. Local recurrence has been approximated as 36% within 5 years with 5-year mortality ~30% [11]. Adjuvant radiation after surgical excision in cases with lymph node involvement has been efficacious in several studies [12]. Several combinations of chemotherapeutic agents have been tried with variable success for aggressive tumors that metastasize or recur despite several wide excisions and radiotherapy [13]. Based on treatment regimens for other head and neck malignancies, chemotherapy regimens for sebaceous carcinoma are classically cisplatin-based and frequently combined with 5-fluorouracil and paclitaxel [13]. Responses in several case reports vary from shrinkage of metastatic lesions to complete response with several years follow up. Our patient was surgically resected with negative margins. He received radiation therapy for locoregional control. There was no local recurrence however he presented with distant metastasis to spine after 6 months for which he received chemotherapy.

## CONCLUSIONS

Malignant adnexal tumor presenting as a sebaceous carcinoma of trunk in a young adult with local lymphnode and distant bone metastasis is rare. It imposes a significant diagnostic quandary with vague presentation and other mimics. Proper diagnostic methods and adequate surgical intervention can ensure good prognosis, despite its propensity to recur and metastasize.

## CONFLICTS OF INTEREST

All the authors declared no potential conflict of interests with respect to the research, authorship and/or publication of this article.

## FUNDING

The authors received no financial support for the research, authorship and/or publication of this article.

## CONSENT FOR PUBLICATION

Written informed consent was obtained from the patient for the publication of this case report and accompanying images.
