# Correction to: Sebaceous carcinoma of trunk with bilateral axillary lymph node metastasis: a rare presentation of malignant adnexal tumor in young adult

**DOI:** 10.1093/jscr/rjac383

**Published:** 2022-08-16

This is a correction to: Lokesh Shekher Jaiswal, Durga Neupane, Nimesh Lageju, Sarada Khadka, Bijay Sah, Anju Pradhan, Sebaceous carcinoma of trunk with bilateral axillary lymph node metastasis: a rare presentation of malignant adnexal tumor in young adult, *Journal of Surgical Case Reports*, Volume 2022, Issue 6, June 2022, rjac280, https://doi.org/10.1093/jscr/rjac280

In the originally published version of this manuscript the third author's name was incorrectly spelt “Nimesh Lagegu”.

This error has now been corrected.

